# Obituary

**DOI:** 10.1080/14686996.2021.1969100

**Published:** 2021-09-17

**Authors:** Kazuhito Hashimoto

**Affiliations:** National Institute for Materials Science stam_office@nims.go.jp


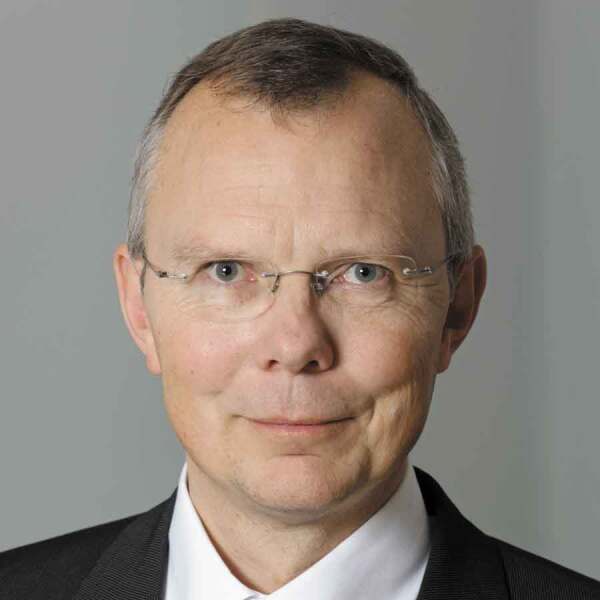


Professor Dr.-Ing Harald Bolt, Advisory Member STAM

We are saddened to learn of the passing of Professor Dr. -Ing Harald Bolt, Advisory Member STAM. Professor Bolt joined STAM’s advisory board in 2015. He was a valuable member of the STAM team, sharing insightful advice based on his extensive research on energy-related materials science.

We would like to express our deepest condolences and thank him for his encouragement and invaluable contributions to STAM.

